# Acquisition of Tigecycline Resistance by Carbapenem-Resistant *Klebsiella pneumoniae* Confers Collateral Hypersensitivity to Aminoglycosides

**DOI:** 10.3389/fmicb.2021.674502

**Published:** 2021-07-02

**Authors:** Hua-le Chen, Yan Jiang, Mei-mei Li, Yao Sun, Jian-ming Cao, Cui Zhou, Xiao-xiao Zhang, Yue Qu, Tie-li Zhou

**Affiliations:** ^1^Department of Clinical Laboratory, The First Affiliated Hospital of Wenzhou Medical University, Wenzhou, China; ^2^Department of Laboratory Medicine, The Second Affiliated Hospital of Wenzhou Medical University, Wenzhou, China; ^3^Department of Infectious Diseases, Sir Run Run Shaw Hospital, Zhejiang University School of Medicine, Hangzhou, China; ^4^School of Laboratory Medicine and Life Science, Wenzhou Medical University, Wenzhou, China; ^5^Biomedicine Discovery Institute, Department of Microbiology, Faculty of Medicine, Nursing and Health Sciences, Monash University, Clayton, VIC, Australia

**Keywords:** collateral sensitivity, CRKP, aminoglycosides, tigecycline, antimicrobial resistance

## Abstract

Tigecycline is a last-resort antibiotic for infections caused by carbapenem-resistant *Klebsiella pneumoniae* (CRKP). This study aimed to broaden our understanding of the acquisition of collateral hypersensitivity by CRKP, as an evolutionary trade-off of developing resistance to tigecycline. Experimental induction of tigecycline resistance was conducted with tigecycline-sensitive CRKP clinical isolates. Antimicrobial susceptibility testing, microbial fitness assessment, genotypic analysis and full-genome sequencing were carried out for these clinical isolates and their resistance-induced descendants. We found that tigecycline resistance was successfully induced after exposing CRKP clinical isolates to tigecycline at gradually increased concentrations, at a minor fitness cost of bacterial cells. Quantitative reverse transcription-polymerase chain reaction (RT-PCR) found higher expression of the efflux pump gene *acrB* (5.3–64.5-fold) and its regulatory gene *ramA* (7.4–65.8-fold) in resistance-induced strains compared to that in the tigecycline-sensitive clinical isolates. Stable hypersensitivities to aminoglycosides and other antibiotics were noticed in resistance-induced strains, showing significantly lowered MICs (X 4 – >500 times). Full genome sequencing and plasmid analysis suggested the induced collateral hypersensitivity might be multifaceted, with the loss of an antimicrobial resistance (AMR) plasmid being a possible major player. This study rationalized the sequential combination of tigecycline with aminoglycosides for the treatment of CRKP infections.

## Introduction

Carbapenem-resistant *Klebsiella pneumoniae* (CRKP) has emerged as one of the deadliest causes of hospital-acquired infections and poses a serious therapeutic concern (Petrosillo et al., 2013). Overproduction of various carbapenemases in CRKP, such as *Klebsiella pneumoniae* carbapenemase (KPC) and metallo-β-lactamases (MBLs), renders the bacteria resistant to nearly all β-lactam antibiotics including carbapenems (Petrosillo et al., 2013).

Tigecycline and colistin are the very few options in the antibiotic arsenal for infections caused by carbapenem-resistant Enterobacteriaceae (Stein and Babinchak, 2013; Sun et al., 2013). Resistance to tigecycline of *Klebsiella* or other Enterobacteriaceae has emerged after its wide use in clinical settings, either as a monotherapy or in combination with other antibiotics (Woodford et al., 2007; Poulakou et al., 2009; Hirsch and Tam, 2010; Sekowska and Gospodarek, 2010; Stein and Babinchak, 2013; Sun et al., 2013; Gonzalez-Padilla et al., 2015). Underlying molecular mechanisms of tigecycline resistance are complex, with the upregulation of resistance-nodulation-division (RND) efflux pumps being a major contributor. Efflux pump systems related to tigecycline resistance include AcrAB-TolC and its positive regulators, *ramA*, SoxS, MarA or its negative regulator r*amR* (Hentschke et al., 2010; Roy et al., 2013; Villa et al., 2014; He et al., 2015; Juan et al., 2016; Xu et al., 2016), OqxAB and its positive regulator *rarA* and negative regulator *oqxR* (Zhong et al., 2014; Juan et al., 2016), and KpgABC (Nielsen et al., 2014; Juan et al., 2016). Structural alteration of the 30S ribosomal subunit protein S10 is another important resistance mechanism, mediated by mutations in the *rpsJ* gene and consequential target site modifications (Villa et al., 2014). A unique enzymatic inactivation mechanism mediated by Tet(X3) and Tet(X4), has also been associated with tigecycline resistance of numerous carbapenems-resistant Enterobacteriaceae (He et al., 2019; Sun et al., 2019).

Development of collateral sensitivity is a widespread evolutionary trade-off that occurs in many Gram-negative and Gram-positive bacteria (Barbosa et al., 2017; Lozano-Huntelman et al., 2020). Only a limited number of studies have examined collateral sensitivity in *Klebsiella pneumoniae*. Du et al. (2018) found that *K. pneumoniae* synchronously regained susceptibility to imipenem and meropenem when the patient developed resistance to tigecycline after long-term antibiotic treatment (Du et al., 2018). Villa et al. (2013) reported a reversion to susceptibility to carbapenems of a CRKP clinical isolate under the pressure of tigecycline (Villa et al., 2013). Proposed mechanisms mediating collateral sensitivity include plasticity of mobile elements in the host bacterium (Stohr et al., 2020), change of global gene expression associated with mutations in resistance genes (Maltas and Wood, 2019), amino acid deletions/substitutions that might result in reduced antibiotic hydrolase activity (Frohlich et al., 2019), and resistance mutations in regulatory genes for efflux pumps (Villa et al., 2013; Barbosa et al., 2017). Membrane-potential-altering mutations may also contribute to reversion of bacterial susceptibility to several unrelated classes of antibiotics, by changing the proton-motive force (PMF) and diminishing the activity of PMF-dependent major efflux pumps (Pal et al., 2015; Furusawa et al., 2018).

Recent emergence of CRKP clinical isolates that are resistant to both tigecycline and colistin, and discovery of a plasmid-mediated colistin resistance gene *mcr-1* highlight the pressing need of developing more effective antimicrobial strategies for CRKP infections (Zhang et al., 2018). This study aimed to establish a comprehensive understanding of collateral sensitivity that accompany the development of tigecycline resistance in CRKP, and to rationalize the combination antibiotic therapy using tigecycline and aminoglycosides for CRKP infections.

## Materials and Methods

### Bacterial Isolates

Fifty CRKP isolates were from patients visiting the First Affiliated Hospital of Wenzhou Medical University and clinically diagnosed with CRKP infections between December 2013 and 2014. These clinical isolates were identified to a species level using the following tests: the Vitek II Identification System (bioMérieux Vitec Inc., Hazelwood, MO, United States) and MALDI Biotyper Identification System (MALDI-TOF MS, BioMérieux, Craponne, France).

### Pulsed-Field Gel Electrophoresis Analysis

Genomic DNA of *K. pneumoniae* clinical isolates was digested with restriction endonuclease *Xba*I (Takara Bio, Dalian, China). DNA fragments were separated in a Pulsed-field gel electrophoresis (PFGE) CHEF-Mapper XA system (Bio-Rad, Hercules, CA, United States) in 0.5 × Tris-borate-EDTA buffer at 14°C and 120 V for 19 h, with pulse times ranging from 5 s to 35 s. PFGE results were analyzed using Quantity One 1-D Analysis 4.6.9 (Bio-Rad, Hercules, CA, United States) Software.

### Antimicrobial Susceptibility Test

Antimicrobial susceptibility of 50 CRKP isolates to 15 first-line antibiotics were initially tested with the Vitek II Identification System (bioMérieux Vitec Inc., Hazelwood, MO, United States) and then confirmed by the CLSI recommended broth microdilution or agar dilution methods. The MICs of tigecycline for *K. pneumoniae* clinical isolates and resistance-induced strains were interpreted using breakpoints recommended by the European Committee on Antimicrobial Susceptibility Testing (EUCAST) criteria (susceptible, ≤1.0 μg/ml; medium, 1.0–2.0 μg/ml; resistant, >2.0 μg/ml) (European Committee on Antimicrobial Susceptibility Testing Steering, 2006). Susceptibility of other antimicrobial agents for *K. pneumoniae* was determined by the agar dilution method according to the Clinical and Laboratory Standards Institute (M100-S24, 2014). *Escherichia coli* ATCC 25922 was used as a quality control.

### *In vitro* Induction of Tigecycline Resistance

Four clinical isolates susceptible to tigecycline (K467, K43, K521, and K428) and one tigecycline-resistant clinical isolate (K537) were used to study tigecycline resistance induction and collateral sensitivity of CRKP. These five clinical isolates were assigned to sequence types by Multilocus sequence typing (MLST), according to the *K. pneumoniae* MLST website.^1^ Tigecycline resistance was induced for the tigecycline-susceptible clinical isolates, by gradually exposing them to tigecycline at serially increasing concentrations at a 24 h interval (1/4 MIC to a higher concentration at which there was no bacterial growth) (Liu et al., 2011).

### Examining General Antimicrobial-Resistance Determinants for Tigecycline Resistance

Polymerase chain reaction (PCR) and sequencing were carried out to examine the presence of common antimicrobial-resistant genes in these 5 CRKP clinical isolates and four resistance-induced strains, including ESBLs genes (*bla*_*CTX–M*__–__1_*bla*_*CTX–M*__–__9_, *bla*_*TEM*_, *bla*_*SHV*_, *bla*_*VEB*_ and *bla*_*PER*_), AmpC genes (*bla*_*CMY*_, *bla*_*FOX*_, *bla*_*MOX*_, *bla*_*DHA*_), carbapenemase genes (*bla*_*KPC*_, *bla*_*SPM*_, *bla*_*IMP*_, *bla*_*VIM*_, *bla*_*GES*_, *bla*_*NDM*_, *bla*_*OXA*__–__23_, *bla*_*OXA*__–__48_), PMQR genes (*qnrA, qnrB, qnrC, qnrD, qnrS, qepA, aac(6′)-Ib-cr, oqxA, oqxB*), and 16S-RMTase genes (*armA, rmtA, rmtB, rmtC, rmtD, rmtE*). The mutation of chromosomal genes *gyrA* and *parC* was also examined. Primer sequences for PCR assays were adapted from other published studies (Doi and Arakawa, 2007; Dallenne et al., 2010; Park et al., 2012) and are available on request. Nucleotide sequences were compared by BLAST to align drug-resistance gene nucleotide sequences^2^.

### Outer Membrane Proteins Analysis

A crude outer membrane fraction of CRKP clinical isolates and resistance-induced strains was obtained by growing the bacteria to a mid-log phase, followed by sonication. Outer membrane proteins (OMPs) were separated by sodium dodecyl sulfate polyacrylamide gel electrophoresis (SDS-PAGE). *K. pneumoniae* ATCC 13883 was used as a control for OMP profiling (Shi et al., 2013). The outer membrane protein encoding genes were further amplified using multiple primer pairs among the 4 paired strains (Zhang et al., 2014).

### Inhibition of Efflux Pumps With CCCP

Efflux pump inhibitor carbonyl cyanide m-chlorophenylhydrazone (CCCP, 10 mg/l) was used to examine the impact of efflux pumps on tigecycline resistance (Zhong et al., 2014). Minimum inhibitory concentrations of tigecycline for CRKP clinical isolates and resistance-induced strains were determined by the broth microdilution method in the presence or absence of CCCP. Four times or greater reduction of tigecycline MICs in the presence of CCCP was adopted as a significant inhibition effect for efflux pumps (He et al., 2015).

### Analysis of *acrR*, *ramR* and *oqxR* Mutations

Efflux pump regulator encoding genes including *acrR*, *ramR* and *oqxR* were amplified by PCR with specific primers (Bialek-Davenet et al., 2011; Lin et al., 2016). The nucleotide sequences of *acrR*, *ramR* and *oqxR* were analyzed using a basic local alignment search tool (BLAST)^3^ with *K. pneumoniae* subsp. pneumoniae MGH 78578 as the reference strain (GenBank accession no. CP000647) (Hentschke et al., 2010).

### Quantitative Real-Time PCR

The relative expression levels of genes encoding efflux pumps AcrB and OqxB and their regulators *ramA* and *rarA* were evaluated using a 7500 Real-Time PCR (RT-PCR) system and *rpoB* as the housekeeping gene (Stein and Babinchak, 2013; He et al., 2015). All experiments were performed in triplicate. The relative expression of the target genes was analyzed using the comparative Ct method (2^–Δ^ ^Δ^ ^*Ct*^) with the Agilent MxPro software.

### Genome Sequencing and Annotation

Whole-genome shotgun sequencing was carried out for 5 CRKP clinical isolates and 4 resistance-induced strains, using a standard run of Illumina HiSeq2000 sequencing using a generating 2 × 100 paired-end libraries (500-bp insert size) sequencing strategy according to the manufacturer’s instructions. Clean reads were assembled into scaffolds using Velvet version 1.2.07. Post-Assembly Genome Improvement Toolkit (PAGIT) was then used to extend the initial contiguous sequences (contigs) and correct sequencing errors. Open reading frames (ORFs) were identified using Glimmer version 3.0. Protein-coding sequences were further BLAST-searched against the Antimicrobial Resistance Database (ARDB).

### Plasmid Analysis

Plasmids of 5 CRKP clinical isolates and 4 resistance-induced strains were analyzed by S1-nuclease pulsed-field gel electrophoresis (S1-PFGE). Plasmid DNA was extracted using a QIAGEN Plasmid Midi Kit (Qiagen, shanghai, China) according to the manufacturer’s instructions, and was separated by electrophoresis in 0.8% agarose at 120 V for 1 h. The plasmids of *E. coli* V517 served as a size marker.

### Data Analysis and Statistical Methods

One-way ANOVA or a non-parametric test (Mann-Whitney *U* test) was carried out to compare two means, depending on the data distribution. Statistical significance was assumed at *p* value of less than 0.05. Data analysis was performed using Minitab 16 software (Minitab, State College, PA, United States).

## Results

### Microbiological Characteristics of CRKP Clinical Isolates

**FIGURE 1:**
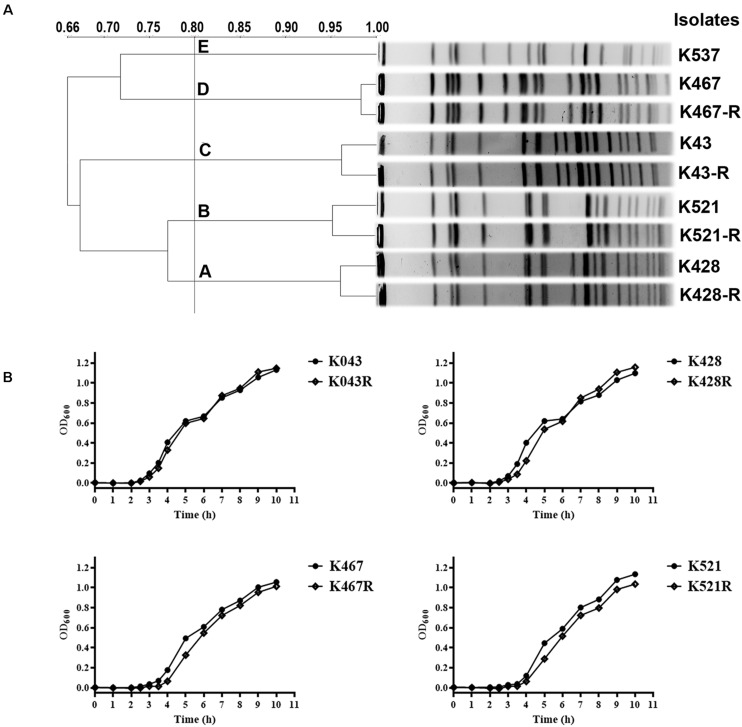
**(A)** Pulsed-field gel electrophoresis (PFGE) analysis of five representative carbapenem-resistant *Klebsiella pneumoniae* (CRKP) clinical isolates and their resistance-induced descendants; **(B)** Growth dynamics of four resistance-induced strains and their parental clinical isolates.

Pulsed-field gel electrophoresis analysis of 50 CRKP clinical isolates showed five clusters, suggesting clonally distinct nature of these isolates (Figure 1A). Antimicrobial susceptibility tests showed that all CRKP isolates were resistant to first-line antibiotics except tigecycline and colistin; one isolate was found to be resistant to tigecycline (K537, MIC 8 mg/L, see Table 1). One representative strain from each cluster (designated as K537, K521, K467, K428, and K43) was chosen for further studies. MLST found that the tigecycline-resistant clinical isolate K537 and tigecycline-sensitive isolates K521, K467, K428, and K43 belonged to sequence types ST39, ST11, ST11, ST37, and ST3623, respectively.

**TABLE 1:** Antimicrobial susceptibility of five CRKP clinical isolates and four resistance-induced descendant strains.

Isolates	Minimum inhibitory concentrations (μg/ml)															
	**AMK**	**CRO**	**CTX**	**CAZ**	**IPM**	**MEM**	**ETP**	**LEV**	**CIP**	**TOB**	**GEN**	**C**	**FOS**	**PB**	**TGC**	**TGC + CCCP**
**K537**	>256	>256	>256	>256	64	128	256	16	64	256	>128	>256	>1024	1	8	4
**K467**	>256	>256	>256	256	128	256	>256	16	64	256	>128	>256	>1024	0.5	0.5	0.5
**K467-R**	<0.25	128	>256	128	64	128	128	32	64	<0.25	0.5	>256	128	0.5	16	4
**K521**	>256	>256	>256	>256	128	256	>256	16	64	256	>128	>256	>1024	1	0.5	0.25
**K521-R**	<0.25	128	256	32	128	128	>256	32	64	<0.25	<0.25	>256	256	1	16	4
**K428**	>256	>256	>256	>256	32	128	>256	16	64	256	>128	>256	>1024	1	0.5	0.5
**K428-R**	0.5	32	8	256	0.25	0.25	0.25	16	64	128	8	>256	4	0.5	16	4
**K43**	>256	>256	>256	64	32	256	256	16	64	128	>128	>256	>1024	1	0.5	0.5
**K43-R**	<0.25	0.25	2	1	0.25	1	8	0.25	1	0.5	1	>256	16	1	32	8

*AMK, amikacin; CRO, ceftriaxone; CTX, cefotaxime; CAZ, ceftazidime; IPM, imipenem; MEM, meropenem; ETP, ertapenem; LEV, levofloxacin; CIP, ciprofloxacin; TOB, tobramycinl; GEN, gentamicin; C, chloramphenicol; FOS, fosfomycin; PB, polymyxin B; TGC + CCCP, tigecycline with CCCP.*

### Experimental Induction of Tigecycline Resistance in CRKP

Four tigecycline-sensitive clinical isolates (K467, K521, K428, and K43) were treated with tigecycline at gradually increased concentrations to induce tigecycline resistance. Significant increases in the MICs of tigecycline (32- – 64- fold) were achieved for the descendant strains in comparison with their parental clinical isolates (Table 1). PFGE analysis further confirmed the origin of the resistance-induced CRKP strains (Figure 1A). The induced tigecycline-resistance remained stable after sixteen passages of CRKP in an antibiotic-free growth medium. Comparing the growth dynamics of tigecycline-sensitive clinical isolates and their resistance-induced descendant strains showed a minor but universal fitness loss (Figure 1B).

### Molecular Mechanisms Underpinning Experimental Induction of Tigecycline Resistance

**FIGURE 2:**
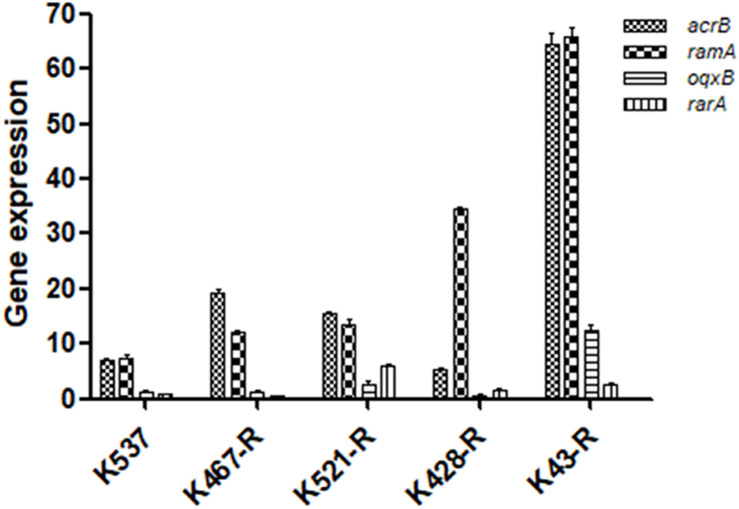
Expression of pump and regulator encoding genes including *acrB*, *ramA*, *oqxB*, and *rarA* in five tigecycline-resistant CRKP strains. The values were expressed as mean ± standard deviation (SD).

We examined the role of adenosine triphosphate (ATP)-driven efflux pumps in the resistance of CRKP to many antibiotics including tigecycline. Using the efflux pump inhibitor CCCP partially restored the susceptibility of all five tigecycline-resistant strains (Table 1). Gene expression of pump- and regulator- encoding genes including *acrB*, *ramA*, *oqxB*, and *rarA* was evaluated (Figure 2). All tigecycline-resistant strains showed higher expression of *acrB* (6. 9-, 19. 3-, 15. 6-, 5. 3-, 64.5-fold for K537, K467-R, K521-R, K428-R, and K43-R, respectively) and its regulator gene *ramA* (7. 4-, 12. 2-, 13. 5-, 34. 5-, 65.8-fold, respectively). Over-expression of *oqxB* and *rarA* was seen in K43-R (12.5-fold and 2.5-fold) and K521-R (2.7-fold and 5.9-fold).

**FIGURE 3:**
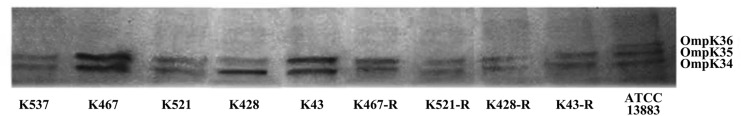
Sodium dodecyl sulfate polyacrylamide gel electrophoresis (SDS-PAGE) of outer membrane proteins (OMPs) in five CRKP clinical isolates and four resistance-induced descendants.

We also sequenced genes encoding other important regulators of efflux pumps, including *acrR*, *ramR*, and *oqxR*. No mutation was identified in *acrR* and *ramR*. Mutations in *oqxR* were found; no difference between tigecycline-sensitive clinical isolates and their resistance-induced counterparts indicated no direct link between *oqxR* mutation and experimentally induced tigecycline resistance (Table 2). SDS-PAGE was carried out to study the role of three major OMPs (ompK34, ompK35, and ompK36) on tigecycline resistance, that may affect the diffusion of tigecycline to its target sites. Using the porin profile of *K. pneumoniae* ATCC 13883 as a control, OmpK36 was missing on SDS-PAGE and amplification of its encoding gene with PCR was unsuccessful (Figure 3), suggesting that a deletion and/or rearrangement of its encoding gene might have occurred in strains with clinical background. Same SDS-PAGE results for tigecycline-sensitive clinical isolates and resistance-induced descendant strains, again suggested no direct association between these OMPs and induced tigecycline resistance.

**TABLE 2:** Mutations of *acrR*, *ramR* and *oqxR* in five clinical isolates and four resistance-induced descendant strains.

Strain no.	*acrR* mutation	*ramR* mutation	*oqxR* mutation
**K537**	No mutation	No mutation	Missense mutation: R152H, M224T
**K43**	No mutation	No mutation	Missense mutation: R152H, M224T
**K43-R**	No mutation	No mutation	Missense mutation: R152H, M224T
**K428**	No mutation	No mutation	Silent mutation: P108P; Missense mutation: R152H, M224T, G317D
**K428-R**	No mutation	No mutation	Silent mutation: P108P; Missense mutation: R152H, M224T, G317D
**K467**	No mutation	No mutation	Silent mutation: P108P; Missense mutation: R152H, M224T, G317D
**K467-R**	No mutation	No mutation	Silent mutation: P108P; Missense mutation: R152H, M224T, G317D
**K521**	No mutation	No mutation	Silent mutation: P108P; Missense mutation: R152H, M224T, G317D
**K521-R**	No mutation	No mutation	Silent mutation: P108P; Missense mutation: R152H, M224T, G317D

### Emergence of Collateral Sensitivity in Tigecycline-Resistant CRPK

Interestingly, prolonged exposure of CRKP to tigecycline reversed their resistance to all tested antibiotics except chloramphenicol and tigecycline; significantly lowered MICs of different antibiotics were found for all resistance-induced descendant strains (Table 1). The level of acquired hypersensitivity seemed to be antibiotic and strain dependent. Up to 2048-fold decreases in the MIC of cephalosporins and carbapenems were observed when tigecycline-sensitive clinical isolates were converted into tigecycline-resistant descendant strains; up to 64-fold and 512-fold decreases in the MIC were found when fluoroquinolones and fosfomycin were tested, respectively. At least 2 out of 4 tigecycline-sensitive and tigecycline-resistant pairs showed relatively small changes in the MIC of these non-aminoglycoside antibiotics (≤4-fold for cephalosporins and carbapenems, ≤2-fold for fluoroquinolones, and ≤16-fold for fosfomycin). More significant decreases in the MIC were noticed for aminoglycosides when compared with other antibiotics (Table 1). Most resistance-induced strains had MICs of amikacin, tobramycin and gentamicin 250-fold lower than that of their parental clinical isolates. The induced hypersensitivity of CRKP to aminoglycosides and other antibiotics remained stable after regrowth in an antibiotic-free medium for 16 passages.

### Evaluating Common Antibiotic Resistance Determinants for Collateral Hypersensitivity of CRKP

Some common antibiotic resistance determinants were examined for 5 CRKP clinical isolates and 4 resistance-induced descendent strains. All isolates harbored the KPC-2 carbapenemase-encoding gene (*bla*_*KPC*__–__2_) and genes encoding other β-lactamases including SHV (sulf-hydryl variable) and TEM (Temoniera in). Genes encoding CTX-M-type ESBLs were only found in the K467 pairs and K521 pairs. Some other common AMR genes conferring antibiotic resistance in CRKP were sequenced, including *gyrA* encoding the DNA gyrase and *parC* encoding DNA topoisomerase that are responsible for fluoroquinolone resistance. An amino acid substitution of S83I was detected in *gyrA* in all 9 strains and S80I was detected in *parC* in the K428, K467, and K521 pairs. *gyrA* mutation D87G was found in the K467 and K521 pairs. The plasmid-carrying quinolone resistance gene *qnrS* was also detected in K537 and K467 pairs. Interestingly, the 16S-rRNA methylase encoding gene *rmtB*, a gene that confers resistance to most clinically relevant aminoglycosides, was found in K467 and K521 but missing in their resistance-induced counterparts.

### Whole Genome Sequencing Broadened Our Understanding of Collateral Sensitivity in CRKP

**FIGURE 4:**
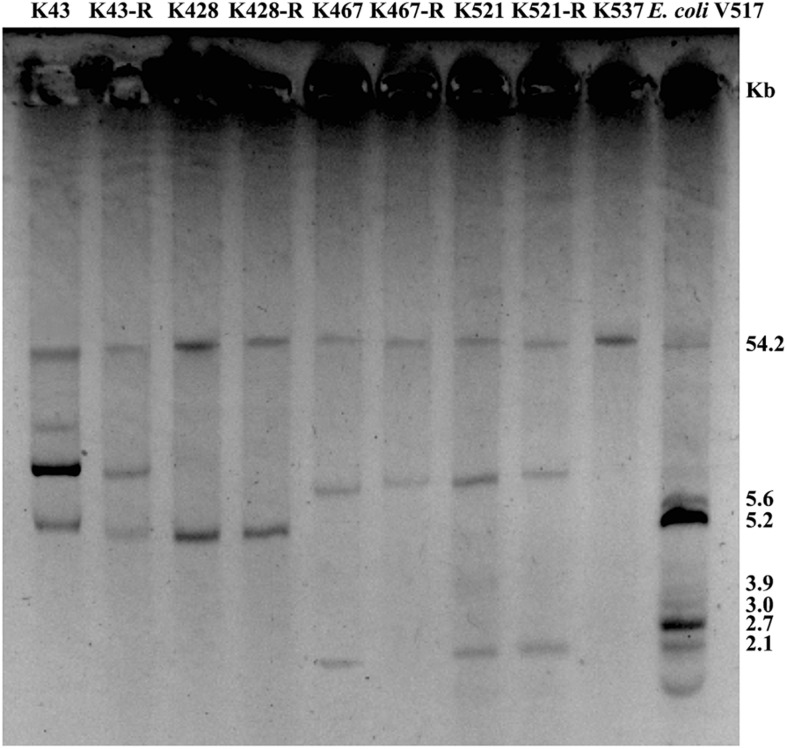
Plasmid analysis of five CRKP clinical isolates and four resistance-induced strains. *Escherichia coli* V517 was used as a control.

Common genetic determinants seemed to be inadequate to explain the induced hypersensitivity to aminoglycosides of CRKP. We thus performed a next-generation full genome sequencing for all nine strains selected for this study, intending to identify molecular determinants that differ between the tigecycline-sensitive and tigecycline-resistant groups. Despite the significant difference in the bacterial resistance to multiple antibiotics including aminoglycosides, no obvious chromosome-related genetic difference was found (Supplementary Figure 1 and Supplementary Table 1, for reviewers’ information only). Full genome sequencing of CRKP revealed several antibiotic-resistance related mutations that were not unique to the tigecycline-sensitive parental strains or their tigecycline-resistant descendants, including: 1) a 4bp mutation in non-coding region between *ybhN* and *ybhL* in the K428 pairs, 2) a 11bp insertion in the non-coding region between *garK-1* and *tRNA_anti-like*, a 10bp insertion in the non-coding region between *kp467_02292* and *kp467_02293* and a 4bp mutation in the gene encoding DNA polymerase IV (*dinB_4)* in the K467 pairs, 3) and a 12bp insertion in the non-coding region between *kp521_05022* and *kp521_05023* in the K521 pairs (Table 3). Several key AMR mobile elements were found in the parental clinical isolates but not the descendants, including *rmtB* (K467 and K521 pairs), *bla*_*TEM*__–__1__*E*_ (K521 pair), *fosA3* (K467 and K521 pairs) (Supplementary Figure 1). As *rmtB* and *fosA3* are often co-carried on a large AMR plasmid, we hypothesized that loss of an AMR plasmid might have occurred to compensate the fitness cost. S1-PFGE revealed that all clinical isolates contained multiple plasmids ranging from 2.1 kb to 54.2 kb in size (Figure 4). One plasmid of 54.2–5.6 kb was absent in K43-R but present in K43, and one plasmid of approximately 2.1 kb was missing in K467-R but not the parental clinical isolate.

**TABLE 3:** Mutations observed in the four clinical isolates and their resistance-induced descendant strains.

Strain	Nearest gene(s)	Reference position (nt)	Reference sequence	Mutant sequence	Mutation type	Predicted effect
**K428/K428-R**	ybhN and ybhL	171782	GGGC	AAAT	MNV	Non-coding region
**K467/K467-R**	*garK-1* and *tRNA_anti-like*	149888	–	GCTATACCAAA	Insertion	Non-coding region
	*kp467_02292* and *kp467_02293*	1318	–	CGAAAAATTT	Insertion	Non-coding region
	*dinB_4*	1703	GGAA	ATCC	MNV	DNA polymerase IV
**K521/K521-R**	*kp521_05022* and *kp521_05023*	1318	–	CGAAAAATTTTT	Insertion	Non-coding region

## Discussion

Extended spectrum beta lactamase-producing CRKP are now among the most critical pathogens advised by the recent WHO global priority list of antibiotic-resistant bacteria (WHO, 2020). The overuse of tigecycline and colistin in clinical settings has promoted CRKP to develop resistance to both last-resort antibiotics (Zhang et al., 2018), highlighting a pressing clinical need for alternative strategies such as antibiotic combination therapy. This study intended to increase our understanding of collateral sensitivity in CRKP that tolerates tigecycline treatment, and such knowledge might serve as the foundation of a promising combination therapy for otherwise “untreatable” CRKP infections.

Carbapenem-resistant *Klebsiella pneumoniae* isolates used in this study have distinct PFGE patterns and belong to different sequence types, supporting the non-dominant clonal nature of CRKP encountered in a hospital environment (Chiu et al., 2017). Development of resistance or non-susceptibility to tigecycline has been linked to prolonged use of tigecycline or other antibiotics (Lin et al., 2016). Our mechanistic study of tigecycline resistance only found an association between the reduced tigecycline susceptibility and the overexpression of *acrB*/*ramA* in CRKP. No significant difference in the expression of *oqxB* and *rarA*, or missense/silent mutations in *oqxR*, *acrR* or *ramR* was found when comparing tigecycline-sensitive clinical isolates and their resistance-induced descendants.

Enterobacteriaceae is known to be able to regain collateral sensitivity to many first-line antibiotics when developing resistance to tigecycline, in particular aminoglycosides (Fang et al., 2016; Barbosa et al., 2017). The underlying mechanisms of collateral sensitivity have been proposed but not been fully disclosed (Barbosa et al., 2017). Barbosa et al. (2017) reported that resistance mutation in the regulatory genes of efflux pumps such as *nalC* or *mexZ* might resensitize β-lactam-adapted bacterial populations to aminoglycosides (Barbosa et al., 2017). Previously reported collateral sensitivity in *K. pneumoniae* was supported by mild decreases in MICs of several first-line antibiotics, by 2–10 times (Fang et al., 2016). We, however, found dramatic changes in MICs of aminoglycosides (>100 times) for all 4 experimentally induced tigecycline-resistant strains. Current mechanisms of aminoglycoside resistance do not seem to sufficiently explain the much greater level of collateral sensitivity of CRKP found in this study (Wachino et al., 2020). We hypothesized that accumulative mechanisms drove the development of collateral hypersensitivity of tigecycline-resistant CRKP. We assessed such trade-offs through whole-genome sequencing of clinical isolates and experimentally induced resistant strains. Genomes of our clinical isolates and resistance-induced descendent strains have high similarity to CRKP clinical isolates of different ST (ST15, ST512, ST11 and ST17) sequenced by others (Hua et al., 2014; Villa et al., 2014; Xu et al., 2017; Du et al., 2018); no major chromosome-related genetic difference were found (Fang et al., 2016). We did find loss of a major AMR plasmid in at least two laboratory evolved strains (K43-R and K467-R). It has been proposed that the co-carriage of various important AMR determinants such as *bla*_*KPC*__–__2_, *bla*_*TEM*__–__1_, *bla*_*CTX–M*__–__14_, and *rmtB* on one plasmid could provide evolutionary benefits to bacteria in an antibiotic-rich environment (Sheng et al., 2012). *K. pneumoniae* is an opportunistic pathogen that is well-known for its diversity of antibiotic resistance genes, with significantly more varied DNA composition, and higher plasmid burden than other Gram-negative pathogens (Wyres and Holt, 2018). The high burden of plasmid carriage in *K. pneumoniae*, however, may result in a comparatively lower fitness of the bacterium (Wyres and Holt, 2018). The complex bacterium-plasmid interaction results in not only the adaption of the host to plasmid, but also specific adaption of plasmid to the host. We thus reasoned the voluntary loss of an AMR plasmid under the long-term pressure of tigecycline might also offer survival advantages to the bacterium (Sheng et al., 2012).

Limitations of this study include that only a small number of isolates were used and the structure of the AMR plasmids involved in collateral sensitivity was not further investigated, due to the limited time frame of this study. Another evident limitation is that tigecycline-resistant clinical isolate failed to show any sensitivity to aminoglycoside or other antibiotics and did not provide direct support to the proposed theory. However, theory raised from this experimental study was supported by findings of several recent clinical studies. Stohr et al. (2020) reported loss of the KPC-gene carrying plasmid and plasmid recombination in KPC-producing *K. pneumoniae* clinical isolates from two patients in a hospital outbreak (Stohr et al., 2020). Fang et al. (2016) also isolated tigecycline non-susceptible clinical isolates that had a 2–4-fold decreases in the MICs to aminoglycosides relative to its tigecycline-sensitive ancestral isolates (Fang et al., 2016).

## Conclusion

Carbapenem-resistant *Klebsiella pneumoniae* may develop collateral hypersensitivity to aminoglycosides and other antibiotics, accompanying the development of bacterial resistance to tigecycline. This experimental study provides a proof of concept to support the sequentially combinational use of tigecycline and aminoglycosides for the treatment of CRKP infections. Antimicrobial susceptibility tests of CRKP should be frequently repeated to provide more specific and accurate guidance for its treatment (Stohr et al., 2020).

## Data Availability Statement

The datasets presented in this study can be found in online repositories. The names of the repository/repositories and accession number(s) can be found below: https://www.ncbi.nlm.nih.gov/, PRJNA706839.

## Author Contributions

YQ and T-LZ: conceptualization and supervision. H-LC, YJ, and M-ML: methodology and validation. H-LC, YJ, and YQ: software. H-LC and M-ML: formal analysis, investigation, and writing—original draft preparation. H-LC, J-MC, and CZ: resources. H-LC and YJ: data curation. CZ, X-XZ, and YQ: writing—review and editing. YS, T-LZ, and YQ: funding acquisition. All authors have read and agreed to the published version of the manuscript.

## Conflict of Interest

The authors declare that the research was conducted in the absence of any commercial or financial relationships that could be construed as a potential conflict of interest.

## Footnotes

1 https://bigsdb.pasteur.fr/klebsiella/klebsiella.html

2 http://blast.ncbi.nlm.nih.gov/Blast.cgi

3 http://www.ncbi.nlm.nih.gov/BLAST

## Supplementary Materials

Supplementary Figure 1 Schematic diagram of comparison of chromosome-harboring or plasmid- harboring resistance genes between tigecycline-sensitive and -resistant pairs (K467 and K521 only). The white arrows illustrate the resistance genes among which the red framed arrows indicate the loss of genes in tigecycline-resistant strains.
