# Supplementary figures and images for: Acquisition of Tigecycline Resistance by Carbapenem-Resistant Klebsiella pneumoniae Confers Collateral Hypersensitivity to Aminoglycosides
Source: Front Microbiol. 2021 Jul 2;12:674502. doi: 10.3389/fmicb.2021.674502 (PMC8284424; doi:10.3389/fmicb.2021.674502)

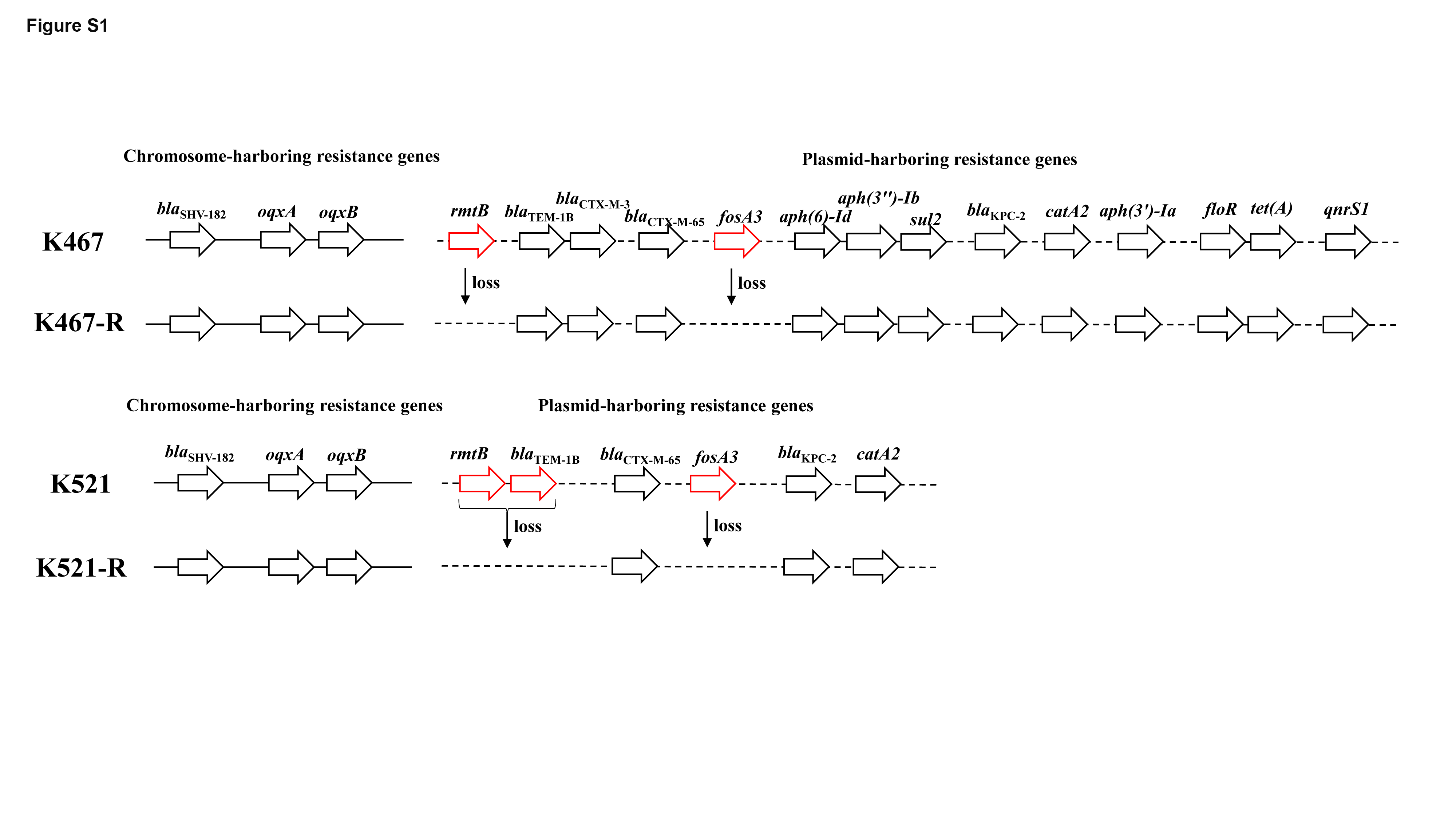

Supplement: Supplementary Figure 1 — Schematic diagram of comparison of chromosome-harboring or plasmid- harboring resistance genes between tigecycline-sensitive and -resistant pairs (K467 and K521 only). The white arrows illustrate the resistance genes among which the red framed arrows indicate the loss of genes in tigecycline-resistant strains. [file Image_1.tif]
