# Supplementary material for: Acquisition of Tigecycline Resistance by Carbapenem-Resistant Klebsiella pneumoniae Confers Collateral Hypersensitivity to Aminoglycosides
Source: Front Microbiol. 2021 Jul 2;12:674502. doi: 10.3389/fmicb.2021.674502 (PMC8284424; doi:10.3389/fmicb.2021.674502)
Supplement: Supplementary file 2 [file Data_Sheet_1.docx]

**SUPPLEMENTARY MATERIAL**

**Table S1. General antimicrobial resistance genes in CRKP clinical isolates and resistance-induced strains.**

| **Isolates** | **Resistance genes** |
| --- | --- |
| **K537** | *bla*_KPC-2_, *bla*_SHV-11_, *bla*_TEM-1_, *qnrS* |
|  |  |
| **K467** | *bla*_KPC-2_, *bla*_SHV-11_, *bla*_CTX-M-9,_ *bla*_TEM-1_, *qnrS, rmtB* |
| **K467-R** | *bla*_KPC-2_, *bla*_SHV-11_, *bla*_CTX-M-9,_ *bla*_TEM-1_, *qnrS* |
|  |  |
| **K521** | *bla*_KPC-2_, *bla*_SHV-11_, *bla*_CTX-M-9,_ *bla*_TEM-1_, *rmtB* |
| **K521-R** | *bla*_KPC-2_, *bla*_SHV-11_, *bla*_CTX-M-9,_ |
|  |  |
| **K428** | *bla*_KPC-2_, *bla*_SHV-11_, *bla*_TEM-1_ |
| **K428-R** | *bla*_KPC-2_, *bla*_SHV-11_, *bla*_TEM-1_ |
|  |  |
| **K43** | *bla*_KPC-2_, *bla*_SHV-11_, *bla*_TEM-1_ |
| **K43-R** | *bla*_KPC-2_, *bla*_SHV-11_, *bla*_TEM-1_’ |

**Table S2. Primer sequences for PCR assays used in this study.**

| Primers | Sequence (5'-3')  (F: forward; R: reverse) | Reference |
| --- | --- | --- |
| *rmtB* | F:GCTTTCTGCGGGCGATGTAA | Doi and Arakawa, 2007 |
|  | R:ATGCAATGCCGCGCTCGTAT |  |
| *rmtC* | F:CGAAGAAGTAACAGCCAAAG | Doi and Arakawa, 2007 |
|  | R:ATCCCAACATCTCTCCCACT |  |
| *armA* | F:ATTCTGCCTATCCTAATTGG | Doi and Arakawa, 2007 |
|  | R:ACCTATACTTTATCGTCGTC |  |
| *rmtA* | F:CTAGCGTCCATCCTTTCCTC | Doi and Arakawa, 2007 |
|  | R:TTGCTTCCATGCCCTTGCC |  |
| *rmtD* | F:CGGCACGCGATTGGGAAGC | Doi and Arakawa, 2007 |
|  | R:CGGAAACGATGCGACGAT |  |
| *bla*_SHV_ | F:GGTTATGCGTTATATTAATC | Park et al., 2012 |
|  | R:TTAGCGTTGCCAGTGCTC |  |
| *bla*_TEM_ | F:ATAAAATTCTTGAAGACGAAA | Park et al., 2012 |
|  | R:GACAGTTACCAATGCTTAATC |  |
| *bla*_CTX-M-1_ | F:AGTTCACGCTGATGGCGACG | Park et al., 2012 |
|  | R:AACCCAGGAAGCAGGCAGTCC |  |
| *bla*_CTX-M-9_ | F:GATTGACCGTATTGGGAGTTT | Park et al., 2012 |
|  | R:CGGCTGGGTAAAATAGGTCA |  |
| *qnrA* | F:AGAGGATTTCTCACGCCAGG | Park et al., 2012 |
|  | R:TGCCAGGCACAGATCTTGAC |  |
| *qnrB* | F:GGMATHGAAATTCGCCACTG | Park et al., 2012 |
|  | R:TTTGCYGYYCGCCAGTCGAA |  |
| *qnrS* | F:GCAAGTTCATTGAACAGGGT | Park et al., 2012 |
|  | R:TCTAAACCGTCGAGTTCGGCG |  |
| *oqxA* | F:CTCGGCGCGATGATGCT | Park et al., 2012 |
|  | R:CCACTCTTCACGGGAGACGA |  |
| *oqxB* | F:ACCGGAACCCATCTCGATGC | Park et al., 2012 |
|  | R:CTCGGCCATTTTGGCGCGTA |  |
| *aac(6’)-Ib-cr* | F:TTGCGATGCTCTATGAGTGGCTA | Park et al., 2012 |
|  | R:CTCGAATGCCTGGCGTGTTT |  |
| *bla*_VEB_ | F:CATTTCCCGATGCAAAGCGT | Dallenne et al., 2010 |
|  | R:CGAAGTTTCTTTGGACTCTG |  |
| *bla*_PER_ | F: GCTCCGATAATGAAAGCGT | Dallenne et al., 2010 |
|  | R: TTCGGCTTGACTCGGCTGA |  |
| *bla*_FOX_ | F:CTACAGTGCGGGTGGTTT | Dallenne et al., 2010 |
|  | R:CTATTTGCGGCCAGGTGA |  |
| *bla*_MOX_ | F:GCAACAACGACAATCCATCCT | Dallenne et al., 2010 |
|  | R:GGGATAGGCGTAACTCTCCCAA |  |
| *bla*_CMY_ | F:CGAAGAGGCAATGACCAGAC | Dallenne et al., 2010 |
|  | R:ACGGACAGGGTTAGGATAGY |  |
| *bla*_DHA_ | F:TGATGGCACAGCAGGATATTC | Dallenne et al., 2010 |
|  | R:GCTTTGACTCTTTCGGTATTCG |  |
| *bla*_GES_ | F:AGTCGGCTAGACCGGAAAG | Dallenne et al., 2010 |
|  | R:TTTGTCCGTGCTCAGGAT |  |
| *bla*_OXA-48_ | F:GCTTGATCGCCCTCGATT | Dallenne et al., 2010 |
|  | R:GATTTGCTCCGTGGCCGAAA |  |
| *bla*_IMP_ | F:TTGACACTCCATTTACDG | Dallenne et al., 2010 |
|  | R:GATYGAGAATTAAGCCACYCT |  |
| *bla*_VIM_ | F:GATGGTGTTTGGTCGCATA | Dallenne et al., 2010 |
|  | R:CGAATGCGCAGCACCAG |  |
| *bla*_KPC_ | F:CATTCAAGGGCTTTCTTGCTGC | Dallenne et al., 2010 |
|  | R:ACGACGGCATAGTCATTTGC |  |
| M = A or C; H = A or C or T; Y = C or T.; R =A or G; S =G or C; D =A or G or T | | |
